# Factors associated with vegetable and fruit intake among adolescents with overweight and obesity in Selangor from 2020 to 2021

**DOI:** 10.3389/fpubh.2025.1539506

**Published:** 2025-07-16

**Authors:** Shaliza A. Shahridzal, May Y. Lau, Ruzita Abd. Talib, Nur Zakiah Mohd Saat

**Affiliations:** ^1^Centre for Community Health Studies (ReaCH), Faculty of Health Sciences, Universiti Kebangsaan Malaysia, Kuala Lumpur, Malaysia; ^2^Nutritional Science Program, Faculty of Health Sciences, Universiti Kebangsaan Malaysia, Kuala Lumpur, Malaysia; ^3^Program of Biomedical Sciences, Centre of Community Health (ReaCH), Faculty of Health Sciences, Universiti Kebangsaan Malaysia, Kuala Lumpur, Malaysia

**Keywords:** vegetables, fruits, prevalence, factors, personal, social-environmental, physical-environmental, adolescents

## Abstract

**Background:**

Substantial scientific evidence firmly advocates consumption of vegetables and fruits for maintenance of overall health and protection against chronic diseases, such as obesity. However, prevalence of fruit and vegetable intake among adolescents in Malaysia remains low, whereas the data on factors associated with vegetable and fruit intake among adolescents were limited.

**Objective:**

This study aims to determine the prevalence of fruit and vegetable intake and the factors that influence the Malaysian adolescents' consumption of fruits and vegetables.

**Methods:**

A cross-sectional study was conducted from November 2021 to August 2022 by distribution of an online validated questionnaire on various platforms to recruit school-going adolescents aged 13 to 17 years old (Form 1–5) in Selangor. Respondents were screened and data of eligible participants were included as subjects. Descriptive statistics, chi square analysis, and generalized linear model with Poisson-loglinear distribution and the robust estimator were employed for data analysis.

**Results:**

A total of 277 adolescents participated in this study. Overall, low prevalence of adequate vegetable consumption was observed (23.5%). Of the participants surveyed, 14.8% of adolescents with thinness, 25.2% and 15.0% of adolescents with overweight and obesity, and 27.1% of normal-weight adolescents met the recommended daily intake (>3 servings), whereas 64.1% of adolescents with overweight and obesity and 65% of normal-weight participants consumed at least two servings of fruits a day. The findings revealed significant association between BMI-for-age (*X*^2^_(1, N = 277)_ = 5.236, *p* = 0.022) and adolescent fruit intake. On the other hand, overweight and obese adolescents reporting positive intention (PR: 1.146, 95% CI: 1.002, 1.310, *p* = 0.047) and parental allowance (PR: 1.125, 95% CI: 1.011, 1.252, *p* = 0.030) were observed to have 14.6% and 12.5% higher prevalence of fruit consumption, respectively, while availability at home (PR: 0.849, 95% CI: 0.731, 0.987, *p* = 0.033) showed significantly lower prevalence of fruit intake with more reports of home availability.

**Conclusion:**

The study suggests that personal, social-environmental, and physical-environmental factors influence vegetable and fruit intake among adolescents, particularly fruit intake consumption behaviors among overweight and obese adolescent population in Selangor. The enunciation of these intake correlates could potentially be incorporated in future development of intervention strategies to effectively promote fruit and vegetable intake.

## 1 Introduction

Adequate consumption of vegetables and fruits is a key strategy in the prevention of obesity. The WHO reported that up to 43% and 16% of the individuals worldwide were either overweight or obese (1). In Malaysia, obesity has been at the forefront of public health issues, with 50.1% of the population either overweight or obese. A high prevalence was also observed among the Malaysian adolescents, with 15.0% of them being overweight and 14.8% of them obese (2). Adequate intake of vegetables and fruits can protect against obesity by counteracting excessive adiposity. Vegetables and fruits do this by increasing bioavailable fibers in the body, which lowers hunger and increases satiety, leading to a decrease in overeating, which is one of the major causes of obesity (3). Another study of the effects of vegetables and fruits against obesity showed a reduction in fat mass composition and body mass index, as well as a 12% decrease in the risk of abdominal obesity among the participants, who have complied with the standard recommendations of vegetable and fruit intake (4). Thus, the importance of adequate vegetable and fruit intake in reducing obesity is further enunciated.

However, despite the known benefits of vegetable and fruit consumption, a low prevalence of vegetable and fruit intake is still observed globally. In 2019, the global prevalence of dietary patterns among adolescents indicated that 34.5% and 20.6% of adolescents have fruits and vegetables less than once a day, a substantial contrast to the recommended five daily servings of fruits and vegetables (5). Similarly, a study conducted in five Southeast Asian countries (Thailand, Myanmar, Indonesia, India, and Sri Lanka) showed that 76% of adolescents aged 13–15 years across all five countries failed to meet the recommended number of servings of fruits and vegetables (6). Similarly, in Malaysia, the prevalence of vegetable and fruit consumption (at least five servings daily) is also extremely low, especially among adolescents, at only 23.5% in the National Health and Morbidity Survey (NHMS) 2017 (7), a drop from the 28.7% prevalence reported in the NHMS 2012 (8). The recent prevalence reported by the Adolescent Health Survey 2022 shows an even lower prevalence, with only 16.1% of adolescents meeting the recommended intake of vegetables and fruits (9).

Studies have emphasized the association of personal, social, and physical-environmental factors with vegetable and fruit intake. Deliens et al. explained that factors such as personal and individualized factors act as key contributors to food decisions, underscoring their significant role in shaping dietary habits. This is due to their close relation to human behaviors shaped from childhood (10). Within the domain of personal factors, evidence has shown several aspects linked to vegetable and fruit intake among adolescents. Rohin et al. (11), in their study in rural Terengganu, found that knowledge, attitude, liking, self-efficacy, intention, and habit were important determinants of vegetable and fruit consumption behaviors. This was further supported by Rahmawati et al. (12) and Hill et al. (13), whereby their studies demonstrated a significantly positive association between knowledge and vegetable and fruit intake. On the other hand, a Thai study has also reported significant associations between positive attitudes toward vegetables and fruits with adequate vegetable and fruit intake (14), while Woodruff (15), Salwa et al. (16), and Moraes et al. (17) reported that determinants such as preference or liking, positive behavioral intentions, and higher levels of self-efficacy toward vegetables and fruits can birth similar results.

Social or social-environmental factors act as another domain closely associated with vegetable and fruit consumption behaviors (11, 18). A network of social relationships is a crucial source of support and seems to have an important impact on health behaviors. This includes relationships and communities that surround the individuals at home, school, workplace, and neighborhood. Substantial evidence supports this notion with evidence of significant associations between vegetable and fruit intake with determinants such as positive parental modelling (18), reinforcing family rules of demand and allows (19), as well as peer influence (20), all can significantly impact the dietary behavior of adolescents toward vegetables and fruits.

On top of that, physical-environmental factors also substantially contribute to the adolescents' consumption behavior toward vegetables and fruits, with emphasis on food availability as the sole most impactful physical-environmental factor in vegetable and fruit consumption. Growing evidence suggests that food availability at home is strongly related to the dietary intake of adolescents. It has been reported that adolescents with lower food availability at home contribute to consuming less fruit (21). Findings from the Family Life, Activity, Sun, Health, and Eating (FLASHE) study revealed that an increased intake of vegetables and fruits would occur when there was a higher availability of vegetables (22). Gustafson and colleagues studied the direct and indirect impacts of food purchasing behaviors, as well as home, school, and consumer food environments, on dietary intake among rural adolescents (23).

The prevalence of obesity and overweightness in Malaysia is widespread across the country. According to the NHMS 2022, Selangor ranked as one of the highest for population obesity among all regions of Malaysia (9). The national data revealed that 17.5% of the populations in Selangor were overweight while 12.6 were obese. Koo et al. (24) highlighted that the increasing prevalence of obesity and overweightness in Selangor was also proportional to the spike in other non-communicable diseases. On top of that, fruit and vegetable intake within the region was consistently low for the past several years, with the latest data revealing only 14.3% consuming adequate vegetables and fruits as per the recommendation (9). This hence forth put Selangor as one of the focus areas for public health interventions. Given the escalating prevalence of obesity and NCDs, it is especially crucial to focus on vegetable and fruit intake as a catalyst for change. The factors associated with the consumption of vegetables and fruits should be highlighted in a multi-sectoral effort. This collaborative approach can help design a tailored intervention strategy to address the issues of obesity and promote the consumption of vegetables and fruits among adolescents. This present study aims to support such developments by provided regional data to better tailor future interventions for highest effectiveness.

Moreover, research on vegetable and fruit intake and behaviors among the adolescents in Selangor, especially those who are overweight or obese, is currently limited. In 2020, we found that there is a relatively apparent gap in research focusing on specific factors or predictors that influence fruit and vegetable consumption within this specific population (obese and overweight adolescents) in Selangor. Existing regional data for the past years consistently covered the prevalence of intake among the adolescents in general (7), however there is a notable scarcity of data and information on the factors that contribute to such behaviors, especially within this population. Therefore, given the importance of said data in regional adolescent public health development and intervention, this study aims to bridge this gap by investigating the factors associated with vegetable and fruit intake behaviors among adolescents with overweight and obesity in Selangor. The goal is to contribute to the improvement of the current situation. We hypothesize that a significant association can be found between factors such as personal, social-environmental, and physical-environment, and vegetable and fruit intake among overweight and obese adolescents in Selangor.

## 2 Materials and methods

### 2.1 Research design and population

This study adopted a cross-sectional research design. From November 2021 to August 2022, 9 months were utilized for the completion of this study. The target population of this study, as specified in the inclusion criteria, was school-going Malaysian adolescents between the ages of 13 to 17 living in Selangor, Malaysia. Non-Malaysians and individuals with diet-related or chronic diseases were excluded from the study.

The sample size was calculated using Cochran's single proportion formula for sample size calculation (25). The sample size calculation for this study includes a confidence interval (z) of 95% = 1.96, with an estimated prevalence of the population that consume fruit and vegetables (p) at only 23.5% (7) and a margin of error (e) of 0.05. The sample size calculated amounted to 277 participants.

This study adopted convenience sampling as the sampling method. An online questionnaire (Google form) on vegetable and fruit intake was designed and advertised on various social media platforms (such as WhatsApp, Telegram, Facebook, and Instagram) and aimed to recruit participants in the state of Selangor. Non-Selangor citizens were excluded from this study. Eligible participants' who have signed the consent form were included as part of the study.

### 2.2 Ethical approval

This study was conducted with ethical approval (Code: UKM PPI/111/8/JEP-2022-226) from the Human Research Ethics Committee of Universiti Kebangsaan Malaysia.

### 2.3 Data collection

The main means of data collection was a four-part self-reporting online questionnaire. Part 1 and Part 2 ask for sociodemographic factors and anthropometry measurements. Part 3 and Part 4 ask questions on the frequency of fruit and vegetable intake and the factors that influenced it. This version of the questionnaire was validated by Rohin et al. (11) and De Bourdeaudhuij et al. (26), respectively for the Malay and English version.

*Part 1: Sociodemographic factors*

This section explains the collected data on the adolescents' sociodemographic factors, including age, sex, ethnicity, parents' education level, and household income.

*Part 2: Anthropometry measurements*

The respondents self-reported their body weight and height. Their body mass indexes (BMI) were then calculated and categorized according to the BMI-for-age z-score (BAZ). Respondents were categorized into groups, which included “thin” if their BAZ values were <−2 SD, “normal” if their BAZ values were between ≥ −2 SD and ≤ +1 SD, and “overweight” if their BAZ values were > +1 SD and “obese” if their BAZ values > +2 SD.

*Part 3: Frequency of vegetable and fruit intake*

This questionnaire comprised questions on the frequency and amount of vegetable and fruit intake. There are two separate sections: frequency and amount of intake. For frequency, the participants were asked how often they consumed vegetables and fruits in a week. The response categories were never, 1 day, 2–4 days, 5–6 days, and every day. For amount, the participants were also asked how many servings of vegetables and fruits they consumed per day, with the response categories being No, one, two, three, four and five or more servings. The amount of participants' vegetable and fruit intake was assessed using the Malaysian Dietary Guidelines for Children and Adolescents 2023 (MDGCA 2023) (27) to determine whether they met the recommended servings, which are five servings (three servings of vegetables and two servings of fruits) daily.

*Part 4: Factors associated with vegetable and fruit intake*

This section has questions regarding the personal, social-environmental, and physical-environmental correlates of fruit and vegetable intake, as adapted from De Bourdeaudhuij et al. (26) and Malay version of De Bourdeaudhuij et al.'s questionnaire (26) adapted and validated by Rohin et al. (11). Validation of this questionnaire revealed that the internal consistency and test-retest reliability of the scales range between Cronbach's α values of 0.52 to 0.89 and Intraclass Correlation Coefficient (ICC) values of 0.52 to 0.85, which were comparable between all five countries involved in the testing (26). Permission to use the questionnaires was obtained from both authors. For this study, different components were included to fully encompass the possible domains of the possible associated factors, including personal factors (knowledge, attitude, liking, self-efficacy, intention, and habit), social-environmental factors (parental modelling, peer modelling, parental encouragement, parental demand, and parental allowance), and physical-environmental factors (availability of fruits and vegetables at home, school, friends' homes, and leisure places). The response category for all these questions was in the form of Likert scales: from Fully Agree (+2) to Fully Disagree (−2) and Very Often (+2) to Never (−2).

### 2.4 Data analysis

Statistical Package for the Social Sciences Statistics (SPSS version 28) was used to analyze the collected data. This study employed four main statistical analysis to fulfil the research objectives. Descriptive statistics, including frequency, percentage, mean, and standard deviation, were used to present the demography and characteristic of the study population. Data on adequacy of vegetable and fruit intake was obtained from computing frequency of intake and number of daily servings into new dichotomous variables. Cases with intake of 7 days and 3 or more daily number of servings (two or more for fruit intake) were denoted as 1, while other entries were put as 0, following the Malaysian Dietary Guidelines for Children and Adolescents 2023 (27). Frequencies and percentages were then used to present the prevalence of vegetable and fruit intake within the population. The chi-square test was used to identify the associations between sociodemographic factors such as age, gender, ethnicity, parent's education level, household income status, and BMI-for-Age with vegetable and fruit intake. Generalized linear model (GLM) with Poisson loglinear distribution and the robust estimator was employed to model the adolescent vegetable and fruit intake based on potential associated factors, including personal factors (knowledge, attitude, liking, self-efficacy, intention, and habit), social-environmental factors (parental modelling, peer modelling, parental encouragement, parental demand, and parental allowance), and physical-environmental factors (availability of fruits and vegetables at home, school, friends' homes, and leisure places). These variables were treated as scores of −2 to +2, and included into the model as continuous variables. A *p-value* of <0.05 was applied as the cut-off for significance.

## 3 Results

### 3.1 Sociodemographic factors

Table 1 shows the sociodemographic information of the participants in this study. A total of 277 adolescents aged 13–17 years (mean age and SD) participated in this study. More than half of the participants are male (*n* = 146, 52.7%). In terms of ethnicity, most are Chinese (*n* = 125, 45%) and have parents with at least secondary school education (*n* = 127, 45.8%). Meanwhile, 40.1% of the participants have moderate family income (*n* = 111). Most of the participants were either overweight or obese (*n* = 143, 51.6%), while others had normal BMI or were thin (*n* = 134, 48.4%).

**Table 1:** Sociodemographic characteristics of adolescents (*N* = 277).

**Sociodemographic factors**	**Frequency^1^ (*N*)**	**Percentage^1^ (%)**
**Age**		
13	49	17.7
14	36	13.0
15	63	22.7
16	83	30.0
17	46	16.6
**Sex**		
Male	146	52.7
Female	131	47.3
**Ethnicity**		
Malay	89	32.1
Chinese	125	45.1
Indian	56	20.2
Others	7	2.5
**Parents' education level**		
Primary	34	12.3
Secondary	127	45.8
Tertiary	116	41.9
**Household income status**		
B40	88	31.8
M40	111	40.1
T20	78	28.2
**BMI-for-age**		
Normal/thinness	134	48.4
Overweight/obesity	143	51.6

1 Descriptive statistics was employed to obtain sociodemographic information of the sample.

### 3.2 Prevalence of vegetable and fruit intake

Table 2 shows the prevalence of vegetable and fruit intake by BMI-for-age status. In this study, the overall prevalence of adolescents who met the recommended intake of vegetables and fruit were 23.5% and 57.8%, respectively. A lower percentage of overweight (25.2%) and obese (15.0%) adolescents consumed more than three servings of vegetables. However, the lowest prevalence was observed among thin adolescents (14.8%), while 27.1% of normal BMI adolescents contributed to the overall prevalence. As for fruit consumption of more than two servings, up to 57.8% of all adolescents consumed the recommended amount of fruits, which includes 51.9% of thin adolescents, 50.5% of adolescents with normal BMI, and 64.1% and 65% of overweight and obese adolescents, respectively.

**Table 2:** Prevalence of vegetables and fruits of adolescents by BMI-for-age.

	**Vegetable intake <3 servings/day *N* (%)^1^**	**Vegetable intake ≥3 servings/day *N* (%)^1^**	**Fruit intake <2 servings/day *N* (%)^1^**	**Fruit intake ≥2 servings/day *N* (%)^1^**
Overall	212 (76.5)	65 (23.5)	117 (42.2)	160 (57.8)
**BMI-for-age**				
Thinness	23 (85.2)	4 (14.8)	13 (48.1)	14 (51.9)
Normal	78 (72.9)	29 (27.1)	53 (49.5)	54 (50.5)
Overweight	77 (74.8)	26 (25.2)	37 (35.9)	66 (64.1)
Obesity	34 (85.0)	6 (15.0)	14 (35.0)	26 (65.0)

1 Descriptive statistics was used to determine the prevalence of vegetable and fruit intake.

### 3.3 Association of sociodemographic factors with vegetable and fruit intake

The associations between sociodemographic factors and vegetable and fruit intake of the adolescents are shown in Tables 3, 4. Fisher's exact test was applied when the expected frequencies assumption was violated for ethnicity in Table 4. The findings observed a significant association between sociodemographic factor, BMI-for-age (*χ*^2^_(1, *N* = 277)_ = 5.236, *p* = 0.022), with fruit intake among overall adolescents. However, no significant association was found between sociodemographic factors and vegetable intake of all adolescents. Similarly, when observing the association between all the sociodemographic factors and the vegetable and fruit intake of overweight and obese participants, no significant association was found. Intake specified among overweight and obese participants; no significant association was found.

**Table 3:** Associations of sociodemographic factors with fruit and vegetable intake of overall adolescents.

**Sociodemographic factors**	**Fruit intake^1^**		***χ^2^***	**Vegetable intake^1^**		***χ^2^***
	<**2 servings** ***n*** = **117**	≥**2 servings** ***n*** = **160**		<**3 servings/day** ***n*** = **212**	≥**3 servings/day** ***n*** = **65**	
Age			4.092			3.592
13	24 (20.5)	25 (15.6)		33 (15.6)	16 (24.6)	
14	15 (12.8)	21 (13.1)		27 (12.7)	9 (13.8)	
15	28 (23.9)	35 (21.9)		50 (23.6)	13 (20.0)	
16	28 (23.9)	55 (34.4)		64 (30.2)	19 (29.2)	
17	22 (18.8)	24 (15.0)		38 (17.9)	8 (12.3)	
Gender			1.293			0.005
Male	57 (48.7)	89 (55.6)		112 (52.8)	34 (52.3)	
Female	60 (51.3)	71 (44.4)		100 (47.2)	31 (47.7)	
Ethnicity			0.859			2.567
Malay	40 (34.2)	49 (30.6)		73 (34.4)	16 (24.6)	
Chinese	53 (45.3)	72 (45.0)		91 (42.9)	34 (52.3)	
Indian	21 (17.9)	35 (21.9)		43 (20.3)	13 (20.0)	
Others	3 (2.6)	4 (2.5)		5 (2.4)	2 (3.1)	
Parent's education level			0.245			1.175
Primary	14 (12.0)	20 (12.5)		25 (11.8)	9 (13.8)	
Secondary	52 (44.4)	75 (46.9)		101 (47.6)	26 (40.0)	
Tertiary	51 (43.6)	65 (40.6)		86 (40.6)	30 (46.2)	
Household income status			1.140			0.423
Bottom 40%	34 (29.1)	54 (33.8)		67 (31.6)	21 (32.3)	
Middle 40%	51 (43.6)	60 (37.5)		87 (41.0)	24 (36.9)	
Top 20%	32 (27.4)	46 (28.7)		58 (27.4)	20 (30.8)	
BMI-for-age			5.236^*^			0.195
Normal/thinness	66 (56.4)	68 (42.5)		101 (47.6)	33 (50.8)	
Overweight/obesity	51 (43.6)	92 (57.5)		111 (52.4)	32 (49.2)	

1 Association between sociodemographic and physical characteristics and vegetable and fruit intake of overall adolescents: Chi square analysis

* Significant association between BMI-for-age and vegetable intake: p < 0.05.

**Table 4:** Associations of sociodemographic factors with fruit and vegetable intake of overweight and obese adolescents.

**Sociodemographic factors**	**Fruit intake^1^**		***χ^2^***	**Vegetable intake^1^**		***χ^2^***
	<**2 servings** ***n*** = **51**	≥**2 servings** ***n*** = **92**		<**3 servings/day** ***n*** = **111**	≥**3 servings/day** ***n*** = **32**	
Age			5.274			2.676
13	10 (19.6)	14 (15.2)		16 (14.4)	8 (25.0)	
14	8 (15.7)	13 (14.1)		17 (16.3)	4 (12.5)	
15	15 (29.4)	17 (18.5)		24 (21.6)	8 (25.0)	
16	11 (21.6)	36 (39.1)		38 (34.2)	9 (28.1)	
17	7 (13.7)	12 (13.0)		16 (14.4)	3 (9.4)	
Gender			0.526			0.364
Male	29 (56.9)	58 (63.0)		69 (62.2)	18 (56.3)	
Female	22 (43.1)	34 (37.0)		42 (37.8)	14 (43.8)	
Ethnicity			2.382			2.723
Malay	17 (33.3)	27 (29.3)		37 (33.3)	7 (21.9)	
Chinese	22 (43.1)	35 (38.0)		41 (36.9)	16 (50.0)	
Indian	10 (19.6)	28 (30.4)		29 (26.1)	9 (28.1)	
Others	2 (3.9)	2 (2.2)		4 (3.6)	0 (0)	
Parent's education level			4.991			4.361
Primary	1 (2.0)	12 (13.0)		8 (7.2)	5 (15.6)	
Secondary	26 (51.0)	44 (47.8)		59 (53.2)	11 (34.4)	
Tertiary	24 (47.1)	36 (39.1)		44 (39.6)	16 (50.0)	
Household income status			4.741			1.166
Bottom 40%	11 (23.4)	36 (39.1)		37 (33.3)	10 (31.3)	
Middle 40%	23 (43.4)	39 (34.1)		43 (38.7)	10 (31.3)	
Top 20%	17 (39.5)	26 (27.7)		31 (27.9)	12 (37.5)	

^a^Fisher's exact test was applied.

1 Association between sociodemographic and physical characteristics and vegetable and fruit intake of obese and overweight adolescents: Chi square analysis.

### 3.4 Personal, social-environmental, and physical-environmental factors associated with vegetable and fruit intake

Tables 5, 6 show the associations among personal, social-environmental, and physical environmental factors and the prevalence of vegetable and fruit intake among the adolescent participants, particularly adolescents with overweightness and obesity.

**Table 5:** Personal, social-environmental, and physical-environmental factors associated with vegetable and fruit intake of overall adolescents.

**Factors**	**Vegetable intake^1^**		**Fruit intake^1^**	
	**PR**	**95% CI**	**PR**	**95% CI**
**Personal**				
Knowledge	1.107	0.896, 1.368	0.985	0.892, 1.089
Attitude	1.090	0.858, 1.386	0.988	0.907, 1.078
Liking	0.882	0.730, 1.064	1.118	0.999, 1.252
Self-efficacy	1.125	0.952, 1.329	1.102	1.013, 1.198^*^
Intention	0.931	0.780, 1.111	1.126	1.013, 1.251^*^
Habit	1.067	0.856, 1.330	0.909	0.830, 0.996^*^
**Social-environmental**				
Parental modeling	1.189	0.906, 1.561	0.945	0.838, 1.065
Peer modeling	0.911	0.697, 1.192	0.962	0.857, 1.079
Parental encouragement	0.986	0.680, 1.428	1.028	0.865, 1.221
Parental demand	1.031	0.868, 1.226	1.044	0.955, 1.142
Parental allowance	0.938	0.780, 1.127	1.045	0.958, 1.139
**Physical-environmental**				
Availability at home	1.102	0.829, 1.463	0.969	0.849, 1.106
Availability at school	1.109	0.917, 1.341	0.950	0.876, 1.031
Availability at a friend's house	0.804	0.669, 0.967^*^	0.971	0.883, 1.069
Availability at leisure place	0.755	0.590, 0.967^*^	0.874	0.793, 0.963^*^

Vegetable intake: ^*^p-value <0.05; ^**^p-value <0.001.

Fruit intake: ^*^p-value <0.05; ^**^p-value <0.001.

1 Association between personal, social-environmental, and physical-environmental predictors and vegetable and fruit intake of overall adolescents: generalized linear model (GLM).

* Significant association between availability at school and at leisure place, with vegetable intake: p <0.05.

^*^Significant association between self-efficacy, intention, habit, and availability at leisure place, with fruit intake: p <0.05.

**Table 6:** Personal, social-environmental, and physical-environmental factors associated with vegetable and fruit intake of overweight and obese adolescents.

**Factors**	**Vegetable intake^1^**		**Fruit intake^1^**	
	**PR**	**95% CI**	**PR**	**95% CI**
**Personal**				
Knowledge	1.168	0.845,1.614	1.000	0.886, 1.129
Attitude	1.301	0.897, 1.889	1.023	0.917, 1.142
Liking	0.899	0.689, 1.172	1.013	0.904, 1.134
Self-efficacy	1.232	0.930, 1.631	1.039	0.925, 1.168
Intention	0.887	0.707, 1.113	1.146	1.002, 1.310^*^
Habit	0.951	0.669, 1.352	0.899	0.798, 1.012
**Social-environmental**				
Parental modeling	1.352	0.915, 1.998	0.978	0.833, 1.148
Peer modeling	0.822	0.576, 1.171	0.936	0.807, 1.086
Parental encouragement	1.125	0.687, 1.842	1.118	0.885, 1.412
Parental demand	1.020	0.772, 1.346	1.012	0.905, 1.130
Parental allowance	0.890	0.650, 1.219	1.125	1.011, 1.252^*^
**Physical-environmental**				
Availability at home	1.109	0.685, 1.796	0.849	0.731, 0.987^*^
Availability at school	1.280	0.926, 1.771	0.907	0.804, 1.023
Availability at a friend's house	0.830	0.643, 1.071	0.900	0.780, 1.038
Availability at leisure place	0.794	0.540, 1.166	0.996	0.888, 1.117

Fruit Intake: ^*^p-value <0.05; ^**^p-value <0.001.

1 Association between personal, social-environmental, and physical-environmental predictors and vegetable and fruit intake of obese and overweight adolescents: generalized linear model (GLM).

* Significant association between intention, parental allowance, and home availability with fruit intake: p <0.05.

This study observed significant association between physical-environmental factors with adequate vegetable intake among the overall adolescent sample. Adolescents with positive reports of availability at friend's house (PR: 0.804, 95% CI: 0.669, 0.967, *p* = 0.020) and at places of leisure (PR: 0.755, 95% CI: 0.590, 0.967, *p* = 0.026) both revealed 19.6% and 24.5% lower prevalence of vegetable consumption. Meanwhile, personal and social-environmental factors did not portray any significant influence on the population vegetable consumption.

On the other hand, the study highlighted self-efficacy (PR: 1.102, 95% CI: 1.013, 1.198, *p* = 0.024), and intention (PR: 1.126, 95% CI: 1.013, 1.251, *p* = 0.028) as significant correlates of fruit intake among the general adolescent population, with positive self-efficacy and intention fostering higher prevalence of fruit consumption within the population. In contrast, habit (PR: 0.909, 95% CI: 0.830, 0.996, *p* = 0.41), and the physical-environmental factor, availability at places of leisure (PR: 0.874, 95% CI: 0.793, 0.963, *p* = 0.006), significantly lower prevalence of fruit intake among the general adolescent population, while no significant association were found among social-environmental factors and fruit intake within the overall adolescent sample.

For overweight and obese adolescents, the findings indicated no significant association between the personal, social-environmental, and physical-environmental factors with vegetable intake. However, overweight and obese adolescents reporting positive intention (PR: 1.146, 95% CI: 1.002, 1.310, *p* = 0.047) and parental allowance (PR: 1.125, 95% CI: 1.011, 1.252, *p* = 0.030) were observed to have 14.6% and 12.5% higher prevalence of fruit consumption, respectively, while availability at home (PR: 0.849, 95% CI: 0.731, 0.987, *p* = 0.033) showed significantly lower prevalence of fruit intake with more reports of home availability.

## 4 Discussion

### 4.1 Prevalence of vegetable and fruit intake

The findings of this study revealed a low consumption of fruits and vegetables observed among overweight and obese adolescents in Selangor. From the results, only 23.5% of the adolescent participants have met the recommended daily intake of vegetables, set at equal to or more than three servings per day, as suggested by the Malaysian Dietary Guideline for Children and Adolescents 2023, in line with standards of the World Health Organization (27, 28). In comparison, the national prevalence of vegetable intake, as of 2022, was at 27.1%, which is like the prevalence found in this study (9). In retrospect to both rates, the NHMS 2017 observed a higher percentage of adolescents meeting the vegetable intake recommendation, which had been at 36.0% (7). At the same time, Rohin et al. (11) found that vegetable intake among adolescents in rural Terengganu showed that only 9% had adequate vegetable intake as per the recommendation, which had been substantially lower than that of this study as well as the national prevalence. However, rurality may play a part in these contrasting results. As studies suggest, rurality and sociodemographic factors do affect health literacy (29). Nevertheless, despite the fluctuating rates observed, the findings across these studies suggest that the prevalence of adequate vegetable intake remains low in the country, especially accounting for the national prevalence across the years.

57.8% of the adolescents were reported to have two or more servings of fruit per day, as per the recommendation of the MDGCA 2023 (27). This was significantly higher than the national prevalence of NHMS 2022, which was at 37.3% (9). Meanwhile, the NHMS 2017 reported up to 46.8% of adequate fruit intake among adolescents (7). This suggests that both reports of the national prevalence were lower compared to the findings of this study. Jamaludin et al. (30) found that in Marang and Hulu Terengganu, lower rates of prevalence were observed, with adequate fruit intake among adolescents was 21.7%. Similarly, a study in Ghana reported only 35.7% of adolescents had adequate fruit intake (31). Although this study suggests a higher prevalence of fruit intake of more than 50% among adolescents in Selangor, other studies, as well as the national prevalence, ultimately suggest a low prevalence of adequate fruit intake among adolescents nationally.

Among the participants with overweight and obesity, the findings showed that 25.2% of overweight and 15.0% of obese adolescents had met the recommended serving of vegetables per day, indicating that only a few of the participants achieved the recommended intake suggested in the Malaysian Dietary Guideline for Children and Adolescents (MDGCA) 2023 (27). In NHMS 2017, adequate vegetable intake among overweight and obese adolescents was reported at 9.1% and 5.9%, respectively (7). Similarly, Selamat et al. (32) reported only 3.6% of overweight and obese adolescents had met the recommended intake for vegetables. The current study showed a greater number of adequate vegetable intakes among overweight and obese adolescents as compared to the national prevalence reported in NHMS 2017 (7). Seidu et al. (31), in comparison, revealed a higher prevalence, with 28.7% of overweight and 33.1% of obese adolescents had adequate vegetable consumption, and higher than our study prevalence.

On the other hand, our study reported a total of 64.1% of overweight and 65.0% of obese adolescents to be having adequate fruit intake daily. In contrast, lower percentage of overweight (27.5%) and obese (32.8%) adolescents consumed adequate amounts of fruits were reported in NHMS 2017 (7). Comparable to the findings on vegetable intake, this study showed a higher number of adequate fruit intake among overweight and obese adolescents compared to the percentage reported in NHMS 2017 (7). Seidu et al. (31) also found comparable results in Ghana with 33.0% of overweight and 26.4% of adolescents with obesity have met the recommended intake for fruits. Meanwhile, Selamat et al. (32) also found relatively similar results with only 32.6% of overweight and obese adolescents having adequate intake of fruits.

In contrast, the present study suggests higher intake of fruits among overweight and obese adolescents compared to those of normal weight, which may contradict the general consensus. This could potentially be attributed to societal pressure. For the past several years, a rise in knowledgeable social media influencers (SMI) covering various range of topics including healthy lifestyles and diets has shifted the societal expectations among Malaysian youth (33). It has then led a social contagion mimicking the healthy diet and lifestyle of SMIs, which became prevalent among adolescents, due to their susceptibility to peer influence and social media (34). On top of that, Farhat et al. (35) revealed that adolescents with overweight and obesity are significantly influenced by stigmatization and social comparison, which was seen to have significant associations with social media usage (36). With such trends circulating, it can be postulated that the higher intake of fruits among overweight and obese adolescents could be the result of societal conformity.

Overall, our study shows evidence that the prevalence of vegetable intake among overweight and obese adolescents remains low, reflective of the national prevalence. Meanwhile, the rates for adequate fruit intake were relatively high compared to all the previous studies (7, 31, 32). However, there are generally limited studies which focus on fruit intake as an individual entity, especially among adolescents with overweight and obesity.

### 4.2 Demographic factors and vegetable and fruit intake

The chi-square test revealed no associations between the sociodemographic factors and vegetable and fruit intake. Likewise, when examining the relationship between sociodemographic factors and vegetable and fruit intake among overweight and obese participants, no significant associations were identified. Similar findings were observed in previous research studies, where sociodemographic factors, particularly age, gender, and ethnicity, were not associated with the vegetables and fruit intake of their samples (36–39).

In this context, our findings align with the result from Jongenelis et al. (40), which showed no significant association between sociodemographic factors of age, location, socio-economic position and adequate vegetable intake, however the study instead revealed significant differences in vegetable intake between sexes. In the same study, Jongenelis et al. (40) also revealed that age significantly influence fruit intake among their sample, but the study suggested otherwise for sex, location, and socio-economic position. Albani et al. (41) then suggested that age and sex are not significant influence of vegetable intake, while fruit intake was observed to be influence by age only. This constitutes a relatively similar stand with the findings of Jongenelis et al. (40) for fruit intake, but contrasts with their findings on vegetable intake. The findings also contrast with the present study, where sociodemographic factors do not significantly influence with fruit and vegetable intake (41). In comparison to the present study, Amini et al. (42) suggested that socio-economic status and location positively affects vegetable and fruit intake, contradicting the findings of the present study as well as the of Jongenelis et al. (40).

On another note, parents' education level and household income status were also seen to not have a significant influence on vegetable and fruit intake among adolescents, as reported by Safdar et al. (43) and Drouillet-Pinard et al. (44). However, other studies like Antonogeorgos et al. (45) and Longacre et al. (46) have found that both parents' education level and household income status do have a significant influence on vegetables and fruit intake in adolescents. The studies suggest that adolescents who had parents with higher education levels and higher household income status would have increased consumption of vegetables and fruits, possibly due to the higher health and nutrition literacy that comes with a higher education tier. Moreover, Hamzah et al. (47) showed that a higher level of health literacy in individuals was observed in higher-income households, compared to the other socioeconomic tiers, for up to 53.4% (47). Parents and familial health and nutrition literacy act as a precursor for facilitating healthy eating behaviors among the children of the household. This notion was supported by researchers Chang et al. (48), who highlighted the importance of dietary literacy as a promoter of health status and good eating behaviors of school-age children and adolescents as evidence supported by their research (48).

### 4.3 Physical characteristics and fruit and vegetable intake

On the other hand, this study has found physical characteristics, particularly BMI-for-Age (*p* = 0.022), as a significant factor in fruit intake among adolescents. This study found that individuals with obesity and overweight had higher adequate fruit intake compared to adolescents with thinness and normal weight, revealing a positive association. Similarly, Ham and Kim (49) revealed a significant positive association between fruit intake and BMI among adolescents. However, our findings constitute a different standing from the ISAAC Global Research, which revealed an inverse relationship between BMI and the intake of fruits and vegetables among adolescents in their cohort (50). This notion aligned with the study by Sharma et al. (51) which revealed that adequate intake of fruits was observed to have paradoxical effects, the anti-obesity factor to the prevention of adiposity. The findings of our study may not have reflected this concept, and a higher percentage of overweight and obese adolescents had adequate intake of vegetables and fruits compared to those with normal weight and thinness.

### 4.4 Personal, social-environmental, physical-environmental factors, and fruit and vegetable intake

The findings of this study emphasized several personal, social-environmental, and physical-environmental factors as significant predictors of vegetable and fruit intake. The general adolescent sample found self-efficacy, intention, habit, and availability at places of leisure potentially determining adolescent fruit intake behavior, while vegetable intake of this population was shown to be influenced by availability at a friend's house or at leisure places.

Meanwhile, the overweight and obese adolescent population found factors such as intention, parental allowance, and home availability, associated with the population's fruit consumption, whereas vegetable intake was found not affected by the all potential determinants.

The findings indicate significant influence of personal factors such as self-efficacy, intentions, and habits on adolescent vegetables and fruits consumption. Cross-sectional studies by Rohin et al. (11) and Sato et al. (52) revealed similar findings, with correlations between higher consumption of vegetables with positive attitudes and personality attributes toward vegetable and fruit consumption among Malaysian and Japanese adolescents. This was consistent with the study conducted in Bangladesh and the US, whereby adolescents with greater intentions are more likely to consume vegetables and fruits compared to those with poor intentions (53). Moreover, González-Gil et al. (54) found that female European adolescents with healthy dietary patterns or habits consumed a significantly greater number of fruits. Habits were also considered as a significant predictor of vegetable and fruit intake among UK adolescents. However, the result from this European study has a possibility of cofounders such as health purposes or the awareness of the important effects of a sufficient number of vegetables and fruit consumption. Even so, positive attitude, good intentions and good habits were still suggested as crucial elements to be implemented in the adolescents' dietary practices (55). Contrarily, studies suggested liking as a personal indicator of fruit intake (56). Preferences or liking are personal aspects nurtured through physiological growth such as sensory development and sensitivity transitioning throughout age. A past study emphasized that a child with higher sensory sensitivity toward a certain taste, such as bitterness, has been shown to be less accepting of vegetables. However, it was enunciated that the sensory sensitivity has the potential to decrease throughout the years and thus may increase acceptability toward bitterness and hence can be nurtured to increase vegetable intake (57). This study has also shown a higher intake of vegetables going into adulthood compared to during childhood.

The findings of our study also demonstrated a significant association between social-environmental factors, namely parental allowance, with fruit consumption among overweight and obese adolescents. This is consistent with findings by Fu et al. (58) and Bailey-Davis et al. (19) who revealed that vegetable and fruit intake among adolescents are significantly associated and can be facilitated by positive parent modelling and family rules such as demand and allow, but contrasts with Rohin et al. (11), who reported parental modelling, parental encouragement, parental demand, and parental allowance were not associated with neither vegetable nor fruit intake of the Malay adolescents in rural Terengganu.

In a way similar to Rohin et al. (11), the present study finds parental modeling, parental encouragement, parental demand, and parental allowance were not significantly associated with vegetable. However, parental allowance was found to influence fruit intake among our overweight and obese adolescents. Nevertheless, Rohin et al. (11) postulated that their findings can be attributed to the potential dominance of personal character. This notion was supported by the findings revealed by Deliens et al. (10), which suggested better effectiveness in increasing vegetable intake seen through improvement of personal or individual subject norms. In addition, the increase in autonomous functioning—also a facilitator of self-efficacy—present during adolescence constituted a strive of freedom from dependence on parents, which consequently leads to less time spend with parents and siblings and may diminish familial-motivated decision-making (59, 60).

Vegetables and fruit intake of adolescents have also shown significant association with physical-environmental factors. This study highlighted that greater availability of fruits in different environments would increase adolescents' intake of fruits. The availability of fruits at home appeared as a significant factor in the fruit intake of overweight and obese adolescents. The current outcome is similar to the findings from the Family Life, Activity, Sun, Health and Eating (FLASHE) study, where adolescents' fruit intake was significantly affected by home availability (22). Home availability of fruits also showed a significant association with fruit intake of adolescent girls in a Brazilian study (42). The findings of this study also suggest that the availability of fruits at friends' houses and leisure places as significant predictors of vegetable and fruit intake. In contrast, some findings also found that availability at a friend's houses was not significantly related to the daily fruit intake of school children in European countries (61). However, availability at leisure places was found to be a significant determinant for adolescents' vegetable intake, although not fruit intake, in the study by Rohin et al. (11). The possible explanation for these factors could be due to the time and frequency spent in certain locations, namely home or school, and their fruit consumption mainly depends on the availability of fruits at those places (62). A study by Gustafson et al. (23) and Azeredo et al. (61) also reported findings in contrast to ours, whereby school environments with higher availability of fruits promote the fruit consumption of adolescents compared to those with lower fruit availability.

### 4.5 Limitation

Several limitations have been accounted for in this study. First, due to the COVID-19 pandemic, data collection was carried out using self-reported online questionnaires, which might increase the risk of over- or underreporting the participants' vegetable and fruit intake. It must also be accounted that the use of technology as the primary medium for data collection may skew the demographic representation within the sample, as it favors more tech-savvy individuals and those with internet accessibility. Adolescents with limited access to the internet may not be able to participate and inadvertently be excluded from the sample. However, due to the COVID-19 restrictions enforced by the government such as strict bans on gatherings and temporary shut-down of education institutions, an online data collection was the viable option at that point in time. In turn participants also had to self-report their weight and height, which increases risk of inaccurate BMI estimation.

### 4.6 Suggestion

To counter these limitations, future research should employ physical forms of data collection, through printed questionnaires and guided data collection sessions at the target institutions by trained enumerators and researchers. Anthropometric data should also be collected on-site and be conducted by trained personnel using the right anthropometric equipment such as the SECA weighing scale for measuring body weight and a stadiometer for measuring height.

## 5 Conclusion

This study has demonstrated a low prevalence of vegetable and fruit intake among adolescents in Selangor, with majority of the sample population did not the standard recommendation of vegetable intake, while more than 50% was revealed to have sufficient consumption of fruits. The findings suggested a higher incidence of inadequate vegetable and fruit intake among adolescents with thinness, followed by obese and overweight adolescents. An association was observed between BMI-for-age of overall adolescents and vegetable and fruit intake. However, no association between other sociodemographic factors was found. Similarly, there had been no association observed between all the sociodemographic factors and vegetable and fruit intake among overweight and obese adolescents. The findings also indicated that fruit intake among overweight and obese adolescents was significantly influenced by personal, social-environmental, and physical-environmental factors, respectively, namely intention, parental allowance, and availability at home. However, the factors portrayed no significant association with vegetable and intake. Nevertheless, this study suggests several personal, social-environmental, and physical-environmental factors as significant correlates of adequate fruit and vegetable intake among adolescents, especially overweight and obese adolescents, and has the potential as reliable source of data for tailoring effective future intervention strategies with the aim to enhance the intake of vegetables and fruits among overweight and obese adolescents in Selangor.

## Data Availability

The original contributions presented in the study are included in the article/Supplementary material, further inquiries can be directed to the corresponding author.
